# Presbyopia and Other Eye Conditions in Teachers in Ghana

**DOI:** 10.3390/ijerph16173209

**Published:** 2019-09-03

**Authors:** Charles Koduah, Catey Bunce, Clare Gilbert

**Affiliations:** 1 Vision For A Nation Foundation, Accra GA496, Ghana; 2 Faculty of Life Sciences and Medicine, King’s College London, London SE1 1UL, UK; 3 Department of Clinical Research, London School of Hygiene and Tropical Medicine, London WC1E 7HT, UK

**Keywords:** presbyopia, visual function, Near Activity Visual Questionnaire

## Abstract

The aim of this paper is to assess the eye health needs of school teachers in the Asutifi districts of Ghana. Presenting distance visual acuity was measured in each eye. Those with visual acuity of <6/12 in one or both eyes had subjective refraction. All underwent basic eye examination and near functional vision was assessed for teachers aged ≥35 years using the Near Activity Visual Questionnaire (NVAQ). Teachers with uncorrected presbyopia were given a near correction and NVAQ was assessed again at two weeks. Three hundred teachers were examined with mean (SD) age of 36.5 (9.7) years, 54.3% were male and 6.3% (95% CI: 3.8 to 9.8%) had a presenting acuity of <6/12 in one or both eyes. The estimated prevalence of moderate visual impairment was 0.7% (95% CI: 0.08 to 2.4%). Lens opacities (50%) and refractive error (18%) were the main causes of visual loss. Seventy-five out of 136 (55.1%, 95% CI: 46.6 to 63.4%) of teachers aged ≥35 years were presbyopic, 45.3% (95% CI: 36.9 to 53.7%) of whom had presbyopic correction. Lack of awareness was the major barrier to presbyopic correction. Median Rasch score for teachers given presbyopic correction (*n* = 39) decreased by 60.6% from 46.0 (IQR: 10.7 to 72.8) to 18.1 (IQR: 0 to 58.9) and overall satisfaction with near vision improved at follow up. Prevalence of presbyopia was high, and spectacles improved satisfaction with near vision.

## 1. Introduction

School eye health programs are an efficient way of making eye health accessible to many children worldwide. These programs focus primarily on uncorrected refractive errors, which affect an estimated 12 million children globally [1]. School teachers are often trained to screen children, but little attention has been paid to the eye health of teachers. 

Globally, 1.1 billion people above the age of 35 years are estimated to be living with presbyopia [2] causing significant productivity loss in the working age group [3]. Only a few studies have explored the prevalence of presbyopia among teachers, being 66.4% in Indonesia and 81.3% in Nigeria [4,5]. In Ghana, the prevalence was 68.1% among senior high school teachers, 29.6% of whom were not corrected [6]. 

With an increasing aging population, age-related eye conditions such as glaucoma and diabetic retinopathy (DR) are also likely to affect teachers. Diabetes is estimated to increase by 55% from 382 million in 2013 to 592 million by 2035 in adults aged 20–79 years [7]. In Ghana, the prevalence of diabetes is 6.3% in adults aged 20 years and above [8]. Diabetic retinopathy affects 93 million people worldwide and accounts for 2.6% of all blindness [9,10]. Glaucoma causes irreversible blindness and accounts for 8.5% of global blindness [11]. In Ghana, the overall prevalence of glaucoma is 6.8%, increasing from 3.7% in people aged ≥40 years to 14.6% in those >80 years [12]. Vision loss from these conditions can be prevented by regular eye screening and early detection and treatment. However, in Ghana, the majority of those affected are not aware they have these conditions [12]. 

The work of teachers entails intensive close work to prepare teaching materials and read students’ scripts. Good distance visual acuity is also important to enable them to identify children in the classroom or playground. Uncorrected presbyopia and visual impairment from age-related eye conditions are, therefore, likely to have a negative impact on their ability to teach. 

The aim of the study was to assess the eye health needs of school teachers in the Asutifi districts of Brong Ahafo region of Ghana. The study area has a total population of about 100,000, and has more than 250 schools with over 1000 teachers [13,14]. The objectives were to estimate the prevalence and determine the causes of visual loss among school teachers of all ages by age and gender, to identify the proportion of school teachers of all ages at risk of loss of vision from glaucoma and diabetic retinopathy, to determine presbyopia correction coverage and identify barriers to uptake of correction among school teachers aged 35 years and above, and to assess the impact of presbyopic correction on vision function among school teachers aged 35 years and above using the Near Activity Visual Questionnaire (NAVQ) [15] by comparing baseline scores with scores two weeks after receiving presbyopic correction.

## 2. Materials and Methods 

This was a school-based quantitative cross-sectional study involving teachers. A minimum sample size of 380 was calculated but due to time and resource limitations, convenience sampling based on the size of the school and proximity was used to recruit the 300 teachers who were available and eligible at the time of the visit. Exclusion criteria were a failure to give consent, or visual acuity could not be measured, but this did not apply to any teacher.

### 2.1. Definitions

Visual loss: Presenting visual acuity of less than 6/12 in one or both eyes. The World Health Organization (WHO) categories of visual impairment relate to the visual acuity in the better seeing eye: Not impaired ≥6/12, mild visual impairment (VI) (<6/12 but can see 6/18), moderate VI (<6/18 but can see 6/60), severe VI (<6/60 but can see 3/60), and blind (<3/60).

Presbyopia: Inability to read the N8 optotype with both eyes open at 40 cm with distant correction (if required) and an addition of ≥1.00D improved near vision by at least one line.

Presbyopia correction coverage (PCC): Met need was the proportion of presbyopic teachers who already had spectacles and could read the N8 optotype at a distance of 40 cm. Unmet need was the proportion of presbyopic teachers who required correction but did not have spectacles and near visual acuity improved vision to N8 at a distance of 40 cm with correction. Presbyopia correction coverage (%) was calculated as met need ÷ (met need + unmet need) × 100.

A teacher was said to be at risk of loss of vision from diabetic retinopathy if he or she had a medical history of diabetes. A teacher was said to be a glaucoma suspect if he or she had a vertical cup to disc ratio (VCDR) of more than 0.7 or VCDR asymmetry of more than 0.2 between the two eyes.

### 2.2. All Teachers

Demographic data of all the teachers were recorded, including their diabetic status and family history of glaucoma. 

**Figure 1:**
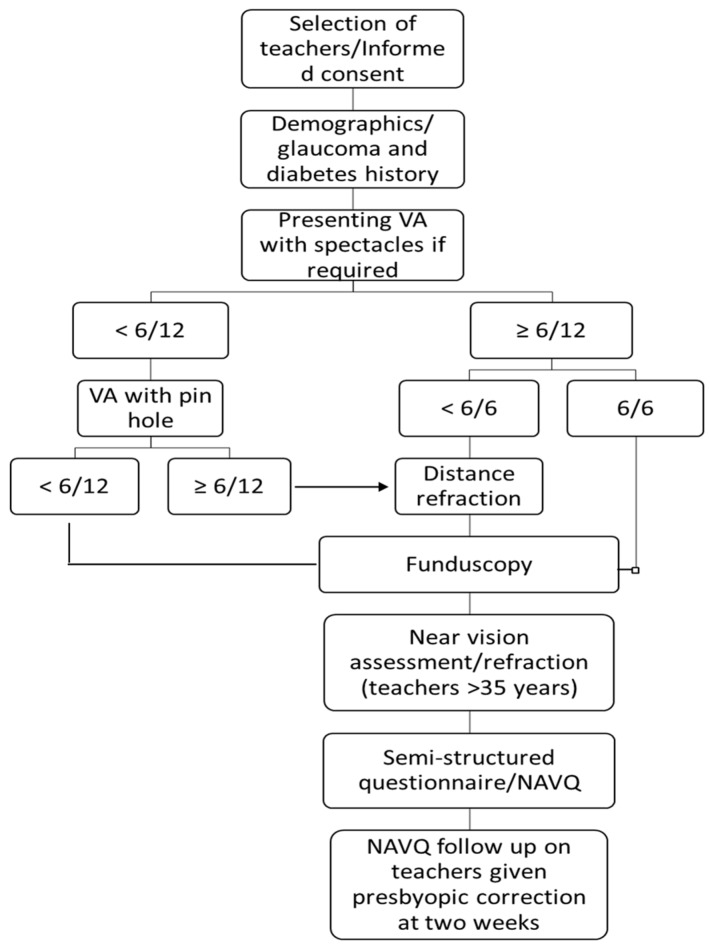
Examination flow chart.

Presenting distance visual acuity (VA) was measured in each eye using a Snellen chart in ambient outdoor illumination at six meters. A teacher was considered to have read a certain line if they correctly identified half of the letters on that line. Teachers who showed improvement in at least one line with a pin-hole underwent distance refraction by a qualified optometrist. Subjective distance refraction was conducted using a trial lens set. All teachers underwent a basic eye examination using a torch light, with undilated fundoscopy with a direct ophthalmoscope. The cause of visual loss was determined for teachers who presented with VA <6/12 in one or both eyes (Figure 1). 

### 2.3. Teachers Aged 35 Years and Above

Presenting near VA was tested binocularly using a near reading chart (Printer’s Point N5 to N24). The chart was held at eye level at 40 cm with the best distant correction, if required. Teachers who presented with a visual acuity of 6/6 were assumed to be emmetrope and were only assessed for near vision. For teachers unable to read the N8 optotype, spherical plus lenses were added in a trial frame in steps of +0.50 dioptersuntil they could see the N8 comfortably at 40 cm and further plus lenses blurred N8 print. Near vision spectacles were given to teachers with uncorrected presbyopia at no cost. 

Teachers who required distance correction or bifocals, or who were at risk of glaucoma or diabetic retinopathy or who had lens opacities were referred to the district hospital. 

A validated near vision visual function questionnaire, NAVQ [15] was administered to teachers aged 35 years and above. In addition to the 10 questions in the NAVQ, school teachers were also asked to rate their overall satisfaction with their near vision on a scale of zero to four, where zero was completely satisfied, and four was completely unsatisfied.

### 2.4. Follow up at 2 Weeks

Teachers who were given presbyopic correction were followed up after two weeks and re-administered the NAVQ.

### 2.5. Data Management

Data were entered into a database created in Microsoft Access and exported to STATA 14 (StataCorp LP., College Station, TX, USA) for analysis. Summary statistics were computed for all baseline variables. Means and standard deviations are reported for normally distributed continuous data, and medians and interquartile ranges for non-normally distributed continuous data. Categorical data are reported as frequencies and percentages. Normality was assessed by inspection of a histogram since the methods used require approximate normality only. Association between categorical variables was assessed using Chi-square tests. The summed NAVQ raw scores for the 10 questions were converted into a Rasch score ranging from 0 to 100 with higher scores indicating greater difficulty with near vision. The Wilcoxon signed rank test was used to assess the significance of observed difference between the median score at baseline and the median score at follow up. All tests were two-tailed, and the level of statistical significance was 0.05.

### 2.6. Ethical Approval

Ethical approval was granted by the research ethics committee, London School of Hygiene and Tropical Medicine (ref: 12109) and the Committee on Human Research, Publication and Ethics in Ghana (ref: CHRPE/AP/126/17). Each teacher provided a written informed consent.

## 3. Results

Three hundred teachers from two senior high schools and fifteen primary schools with a mean (SD) age of 36.5 (9.7) years (range 21–62 years) were examined. There were more males 163 (54.3%) than females. Most of the teachers (55%) were graduates and the others held diploma certificates. One hundred thirty-six (45.3%) teachers were aged 35 years and above (Table 1).

### 3.1. Prevalence and Causes of Visual Loss

Overall, 281 (93.7%) of the teachers had a visual acuity equal to or better than 6/12 in both eyes. Nineteen (6.3%, 95% confidence interval (CI): 3.8–9.8%) had visual loss (presenting VA <6/12 in one or both eyes). More males (15) than females (4) had visual loss in one or both eyes, and lens opacities and refractive error were the main causes (Table 2). There were more males with lens opacities than females (five vs. two). None of the teachers with lens opacities had visual loss severe enough to warrant cataract surgery and all were advised to go for regular reviews at the eye clinic. Seven (2.3%) teachers were prescribed distance correction and one with a pterygium was referred for excision. 

### 3.2. Prevalence of WHO Visual Impairment

Two hundred ninety-five teachers demonstrated normal acuity, three had a mild visual impairment (VI) and two had moderate VI. The estimated prevalence of moderate VI was 0.7% (95% CI: 0.08%–2.4%). All three teachers with mild VI were male whilst there was a single teacher of each gender with moderate VI. Two of the teachers with mild VI, aged 55 and 58 years, had lens opacities, the other had a refractive error. One of the teachers with moderate VI, aged 59 years had lens opacities, the other had no cause recorded.

### 3.3. Risk of Vision Loss from Glaucoma and Diabetic Retinopathy

Five teachers (1.7%) reported to have been diagnosed with glaucoma but only two were taking treatment. Six further teachers (2.0%) were suspected of having glaucoma based on their VCDR. All 11 (3.7%) teachers were referred for glaucoma assessment. Two teachers were diabetic with an average duration of ten years, but neither had signs of diabetic retinopathy. These teachers were advised to go for an annual eye examination at the eye clinic.

### 3.4. Presbyopia Correction Coverage and Barriers to Uptake of Correction

Seventy-five out of 136 teachers aged ≥35 years were presbyopic, giving a prevalence of 55.1% (95% CI: 46.6–63.4%). Presbyopia was significantly associated with age (*p* < 0.001): 57/61 teachers who were not presbyopic were under 50 years compared with 35/75 of those who were presbyopic. 

**Figure 2:**
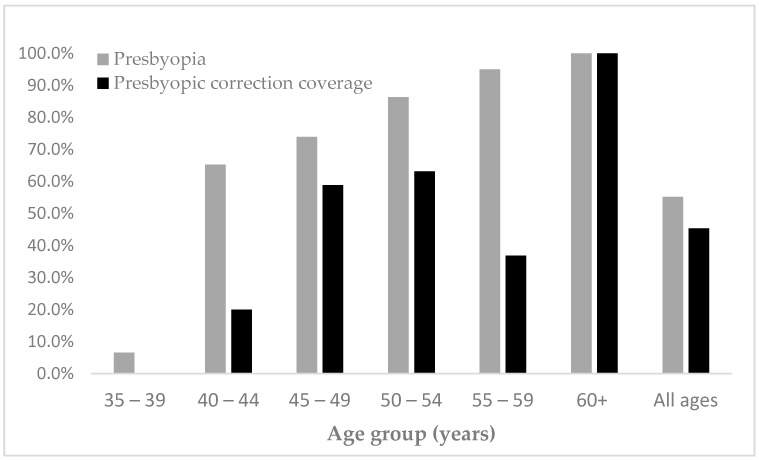
Prevalence of presbyopia and presbyopic spectacle coverage among teachers aged 35 and above, by age.

Thirty-four teachers already had correction whilst 41 did not, giving a presbyopic correction coverage of 45.3% (95% CI: 36.9–53.7%) with an unmet of 54.7%. 21/42 (50%) presbyopic males had correction compared with 13/33 (12%) females. This was not statistically significantly different. Graduate teachers (67.6%) had the highest met need, and coverage tended to increase with age (Figure 2).

**Figure 3:**
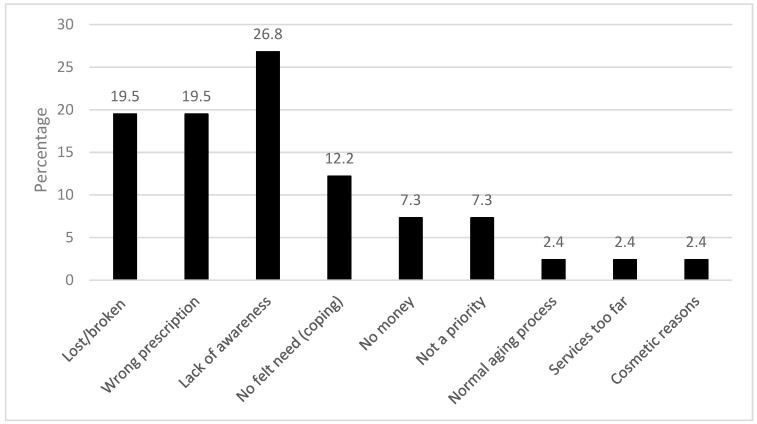
Barriers to presbyopic correction coverage.

The most frequent reason for lack of presbyopic correction was lack of awareness, particularly among males (8/11). Several teachers reported that they had lost their spectacles, or they were broken, or they had the wrong prescription. Other reasons were ‘no felt need’ or did not see it as a priority (Figure 3).

### 3.5. Impact of Presbyopic Correction on Visual Function

One hundred thirty-six teachers aged 35 years and above were administered the NAVQ at baseline. Thirty-nine presbyopic teachers with an unmet need who were given presbyopic correction (range +1.00DS to +3.50DS) were followed up after two weeks. Median Rasch scores at baseline and follow up were compared. 

The median NVFQ score of all 136 teachers at baseline was 23.1 (interquartile range (IQR): 0–43.6). The 61 non-presbyopic teachers had better near visual function (i.e., lower scores) than the 75 presbyopic teachers (median scores 0, interquartile range (IQR) 0–18.3 and 36.1, IQR: 23.07–52.73, respectively). The near visual function of teachers with presbyopia correction was better than for those without, with an NVFQ score ratio of 2:1 (met need: 23.1, IQR: 0–41.2, unmet need 46.0 IQR: 33.3–56.89).

Before being given a near correction, the median score for the 39 teachers was 46.0 (IQR: 33.3–56.89). Females were more affected by near VI than males, with scores of 52.7 (IQR, 10.7–70.6) and 41.2 (IQR: 30.3–72.8), respectively. At follow up near visual function had increased significantly, reflected by a 60.6% decline in their median scores to 18.1 (IQR: 0–36.1) (*p* < 0.001), with similar improvements in males and females.

At baseline 2.6% of the presbyopes with an unmet need reported that they were completely satisfied with their near vision, while 30.8% were very satisfied, 56.4% were a little satisfied, and 10.3% were completely unsatisfied. Two weeks after correction, 53.9% were completely satisfied, 25.6% were very satisfied, 20.5% were a little satisfied and none were unsatisfied. These differences were statistically significant (*p* < 0.001).

## 4. Discussion

In this study, one in fifteen teachers had a visual acuity of less than 6/12 in one or both eyes, and less than 1% were visually impaired. Using WHO categories, no teachers presented with severe visual impairment or blindness. This may be due to the nature of teaching as it demands good near and distant vision, and therefore, people with severe visual impairment are unlikely to become or remain in the profession. This means that prevalence estimates in this study cannot be compared to population-based prevalence surveys in which representative samples are selected, due to the likely selection bias in our sample of teachers. In the Ghana National Blindness and Visual Impairment Study (GNBIS) the prevalence of blindness and severe visual impairment among all ages were 0.74% and 1.07%, respectively [16]. The main causes were cataract and refractive error, as in this study and in global estimates. In terms of gender distribution, there were more males with visual impairment and a similar pattern was found by the GNBIS. 

In this study, several teachers had early lens opacities, two of whom had a mild visual impairment. Although these two teachers were relatively young, risk factors for lens opacities were not explored in this study. None of the teachers required cataract surgery, which in Ghana is usually performed once the visual acuity declines to 6/60 or less, but all were referred. 

This study supports the findings that presbyopia occurs at an early age in Africa [5,17,18] as 4% of the presbyopic teachers were 35–39 years of age. The prevalence of presbyopia was lower than the 68.1% reported among senior high school teachers in Ghana [6]. However, senior high school teachers are likely to be older than teachers in lower levels of education, as in this study. The prevalence was also lower than in school-based studies in Nigeria [5] and Indonesia [4] where a large proportion of the teachers were older than in the present study.

Presbyopic correction coverage (PCC) among teachers in our study was similar to that in Indonesia where 41% had correction [4] but lower than in studies in Ghana and Nigeria where 70.4% and 61.5% of teachers were corrected, respectively [5,6]. In this study, half of the males had correction compared with approximately a third of female teachers even though they have the same or similar salaries. Higher PCC amongst males was also reported in a population-based study in Tanzania where males reported that correction improved employment opportunities [19]. Other reasons, such as cultural beliefs may also contribute to gender differences.

Cost was not the major barrier to having presbyopic correction, as has been reported in several population-based studies in Africa and Asia [18,20,21], but lack of awareness, which was also reported by teachers in Nigeria [5]. This was not expected as teachers are constantly involved in near work and would, therefore, be likely to detect any change in near vision. One explanation is that they had subconsciously adapted to milder degrees of presbyopia. Several teachers had not replaced spectacles that were lost, broken or their spectacles had the wrong prescription but reasons for this were not specifically sought. 

This study has shown that overall satisfaction of near vision function among uncorrected presbyopes can be greatly improved by appropriate corrective lenses. After two weeks of correcting their difficulty with near vision, all 39 teachers were satisfied to varying degrees, with no reports of dissatisfaction which was reported by 10.3% of teachers at baseline. The 60.6% reduction in NAVQ scores also demonstrated that the majority of teachers had their near vision improved by spectacles. A similar improvement was observed in Tanzania where threading a needle was used as a performance-based test [19].

The sample size was relatively small, but the study was adequately powered to detect a statistical difference in the proportion of male and female teachers who were presbyopic but was not large enough to detect differences in spectacle coverage by sex. In addition, the convenience sampling used may have led to a sample of teachers which was not representative of all teachers in the region. The two weeks of follow up to assess the impact of presbyopic correction were not adequate as it is possible that some teachers may not have engaged in much near activity within this period and may not have used the spectacles long enough to appreciate change. In assessing the risk of loss of vision from glaucoma and diabetic retinopathy, the researcher relied on self-report by the teachers, and a dilated retinal examination was not undertaken for those known to be diabetic. Data were also not obtained from the referral eye clinic to assess uptake of referral and whether glaucoma was diagnosed amongst those referred as glaucoma suspects.

## 5. Conclusions

The current study has shown the need to incorporate eye screening of teachers into school health programs in Ghana and elsewhere, and to increase awareness about the causes and management of presbyopia and other eye conditions. The improvement in the overall satisfaction with near vision with presbyopic correction can be used to advocate for refraction services and distribution of affordable spectacles for school teachers above 35 years of age. These programs can also be used to identify teachers who may be at risk of diabetic retinopathy, glaucoma, and other sight-threatening conditions and refer them to an appropriate health facility for assessment and early treatment to prevent sight loss. The screening of teachers would also help in raising the overall awareness of eye health throughout the school and community, which may, in turn, benefit many children in schools and their families. 

## Figures and Tables

**Table 1:** Characteristics of the study sample.

Characteristic	*N* (Total = 300)	(%)
Age group (years)		
20–24	7	2.3
25–29	81	27
30–34	76	25.3
35–39	46	15.3
40–44	23	7.7
45–49	23	7.7
50–54	22	7.3
55–59	20	6.7
60+	2	0.7
Gender		
Male	163	54.3
Female	137	45.7
Qualification		
Degree (SSSCE */WASSCE **)	25	8.3
Diploma	110	36.7
Graduate	165	55
Level teaching		
Nursery	24	8
Primary	111	37
Junior high school	78	26
Senior high school	87	29

* SSSCE—Senior Secondary School Certificate Examination ** WASSCE—West African Secondary School Certificate Examination.

**Table 2:** Prevalence and causes of visual loss (presenting visual acuity (VA) <6/12 in one or both eyes).

Cause of VA <6/12	Frequency	%
Lens opacities	7	37
Refractive error	2	10
Macular scar	3	16
Retinal changes	2	11
Pseudophakia	2	11
Pterygium	1	5
Squint	1	5
Not stated	1	5
Total	19	100
